# Wearable Ring-Shaped Biomedical Device for Physiological Monitoring through Finger-Based Acquisition of Electrocardiographic, Photoplethysmographic, and Galvanic Skin Response Signals: Design and Preliminary Measurements

**DOI:** 10.3390/bios14040205

**Published:** 2024-04-20

**Authors:** Gabriele Volpes, Simone Valenti, Giuseppe Genova, Chiara Barà, Antonino Parisi, Luca Faes, Alessandro Busacca, Riccardo Pernice

**Affiliations:** Department of Engineering, University of Palermo, Viale delle Scienze, Building 9, 90128 Palermo, Italy; gabriele.volpes@unipa.it (G.V.); simone.valenti@unipa.it (S.V.); giuseppe.genova@unipa.it (G.G.); chiara.bara@unipa.it (C.B.); antonino.parisi@unipa.it (A.P.); luca.faes@unipa.it (L.F.); alessandro.busacca@unipa.it (A.B.)

**Keywords:** wearable health devices (WHDs), electrocardiography (ECG), photoplethysmography (PPG), galvanic skin response (GSR), oxygen saturation (SpO_2_), pulse arrival time (PAT)

## Abstract

Wearable health devices (WHDs) are rapidly gaining ground in the biomedical field due to their ability to monitor the individual physiological state in everyday life scenarios, while providing a comfortable wear experience. This study introduces a novel wearable biomedical device capable of synchronously acquiring electrocardiographic (ECG), photoplethysmographic (PPG), galvanic skin response (GSR) and motion signals. The device has been specifically designed to be worn on a finger, enabling the acquisition of all biosignals directly on the fingertips, offering the significant advantage of being very comfortable and easy to be employed by the users. The simultaneous acquisition of different biosignals allows the extraction of important physiological indices, such as heart rate (HR) and its variability (HRV), pulse arrival time (PAT), GSR level, blood oxygenation level (SpO_2_), and respiratory rate, as well as motion detection, enabling the assessment of physiological states, together with the detection of potential physical and mental stress conditions. Preliminary measurements have been conducted on healthy subjects using a measurement protocol consisting of resting states (i.e., SUPINE and SIT) alternated with physiological stress conditions (i.e., STAND and WALK). Statistical analyses have been carried out among the distributions of the physiological indices extracted in time, frequency, and information domains, evaluated under different physiological conditions. The results of our analyses demonstrate the capability of the device to detect changes between rest and stress conditions, thereby encouraging its use for assessing individuals’ physiological state. Furthermore, the possibility of performing synchronous acquisitions of PPG and ECG signals has allowed us to compare HRV and pulse rate variability (PRV) indices, so as to corroborate the reliability of PRV analysis under stationary physical conditions. Finally, the study confirms the already known limitations of wearable devices during physical activities, suggesting the use of algorithms for motion artifact correction.

## 1. Introduction

In the last decade, we have witnessed the widespread development and commercialization of novel biomedical technologies, well known as wearable health devices (WHDs), which have captured public interest thanks to their potential as non-invasive tools for health monitoring [1,2]. WHDs are biomedical devices characterized by minimal weight and size, deliberately designed for comfortable wear, and enabling the continuous acquisition of multiple biosignals [3,4]. These attributes make WHDs potentially valuable tools for remote monitoring of both fragile and healthy individuals, being able to be employed in clinical and daily healthcare environments [5,6]. They can assist the healthcare system in assessing physiological functions during convalescence periods [7,8], post-surgical responses to specific therapies [9,10], and even facilitate the early diagnosis of disorders affecting different organs [11,12,13]. In this context, the scientific community is currently engaged in the design and achievement of new integrated sensors allowing the non-invasive concurrent acquisition of different biosignals from the same body district, and in the development of measurement techniques and signal processing algorithms able to extract novel physiological indices capable of providing further information on the health status [14,15,16].

In this regard, photoplethysmography (PPG) [17,18,19] is one of the most widely employed non-invasive measurement methods, allowing the simple and comfortable extraction of key cardiovascular parameters, making it widely implemented in almost all commercially available wearable devices [20,21,22]. The ease of use, non-invasiveness, and low cost of PPG sensors and devices have contributed to its growth and employment. Photoplethysmography has been typically compared with the less comfortable electrocardiographic (ECG) technique, representing the current clinical gold standard for evaluating cardiac functions [23,24,25]. Starting from the pulse wave detected by a PPG sensor, it is indeed possible to obtain the Pulse-to-Pulse interval (PP) [17], defined similarly to the ECG R-R interval (RR) [26]. From PP and RR time series, it is possible to extract various physiological parameters [27,28,29], such as heart rate (HR) and HR variability (HRV), the latter allowing to gain significant insights into cardiocirculatory regulation mechanisms and the autonomic tone in different physiological states, as well as the overall functionalities of the cardiovascular system [30,31,32]. The synchronous acquisition of PPG and ECG signals also allows to extract a further physiological parameter known as pulse arrival time (PAT) [33], which holds great potential in the realm of cardiovascular health assessment. By reflecting the time taken for the pulse wave to travel through the circulatory system, PAT can be utilized for estimating important parameters such as arterial stiffness or even blood pressure [34,35]. Lastly, from a PPG signal, it is possible to evaluate blood oxygen saturation level (SpO_2_) [36], by utilizing two light sources at different wavelengths, and even estimate the respiratory rate by using suitable signal processing techniques, thus enabling the extraction of useful information about the respiratory system functionality [37].

Another significant biosignal is the galvanic skin response (GSR), i.e., the measurement of the electrical conductance offered by the epidermal tissue, whose variations reflect the regulatory mechanisms of the autonomic nervous system (ANS) and, in particular, of the sympathetic nervous system (SNS). When a strong SNS activation occurs, the activity of the sweat glands and, consequently, the sweat produced by the human body increase proportionally [38]. This leads to the GSR signal being considered as another physiological parameter that can be used alongside HRV indices for discriminating physiological resting states. In fact, when a stressful event of any nature (e.g., mental, physical, emotional) occurs, the organism responds with a defense mechanism better known in the literature as General Adaption Syndrome (GAS), in which complex mechanisms of autonomic regulation are orchestrated by the ANS, entailing sudden changes in various physiological parameters (e.g., HR, sweat, respiratory rate) [39].

Finally, it is also interesting to integrate within wearable devices motion sensing technologies, in order to detect subject movements, allowing the identification of motion artifacts, which are among the main sources of signal corruption [40,41]. Additionally, motion sensors can be employed to distinguish between different physiological states, e.g., discerning whether the subject is at rest or engaged in physical activities, as well to detect some dangerous or even pathological conditions, e.g., by allowing fall detection [42] or identifying local tremors that could be indicative of emerging nervous system disorders [43].

The development of wearable devices capable of performing multiparametric acquisitions of the aforementioned parameters is thus of remarkable scientific and commercial interest. The ongoing research is focused not only on the already widely available wrist-worn devices, but also, more recently, on promising smart ring-shaped wearables. Most of devices commercially available (e.g., Fitbit, Galaxy Watch, Oura Ring) [44,45] only allow the extraction of a few cardiovascular and/or respiratory physiological indices, e.g., heart rate, HRV, and SpO_2_ levels. Conversely, certain emerging technologies (such as the Apple Watch [46,47,48]) have demonstrated the potential to integrate ECG sensors directly on the wrist, while other devices (like Circul+) can be worn directly on the fingers, enabling advanced HRV analyses and blood pressure estimation [46,49]. Table 1 lists and compares the main wearable devices available on the market, in terms of design (i.e., wristband, smartwatch or ring), acquired biosignals, sampling rate, extracted features, and computed physiological indices [44,45,46,49]. Nevertheless, studies regarding the reliability of measurements obtained from such devices are still lacking, necessitating further research to validate and promote the use of this promising technology.

In this context, herein, we present a novel ring-shaped wearable device capable of performing the synchronous acquisitions of ECG, PPG, GSR, and motion signals directly on the fingers, starting from a previous prototype of a probe only able to acquire PPG and GSR signals [36]. An initial measurement campaign on healthy subjects using a protocol consisting of rest and orthostatic stress phases has been conducted, in order to test the proper working of the proposed device and to assess the overall quality of the obtained data in typical everyday scenarios, thereby also extracting physiological indices with the aim of evaluating the feasibility to discriminate different physiological states.

## 2. Materials and Methods

### 2.1. Architecture, Design, and Development of the Wearable Ring-Shaped Device

**Figure 1:**
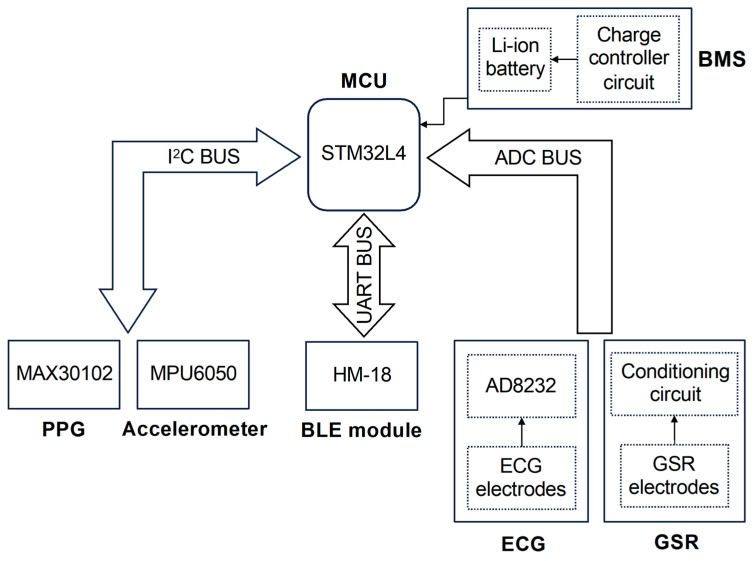
Schematic block representing the architecture of the system. The ultra-low power microcontroller (MCU) handles all the peripherals connected by using three different buses: I^2^C BUS, which allows communications between MCU and digital sensors (PPG and inertial measurement unit); ADC BUS, used to connect analog signals output (ECG and GSR) to the MCU ADC; and UART BUS, which is employed to communicate with BLE module in order to exchange the data collected by the MCU. Lastly, the battery management system (BMS) provides energy to the entire system.

The device proposed in this work has been designed to perform the synchronous acquisition of multiple physiological signals on a single body district, i.e., the fingers, in order to introduce a novel solution exploiting the true potential of wearable technologies. During the design phase, the efforts were focused on developing a compact and comfortable device that employs miniaturized highly integrated components without compromising its lightweight nature and compactness. Our device (whose architecture is depicted in Figure 1) is microcontroller-based, wherein the microcontroller serves as the core of the system, overseeing and managing both analog and digital modules, the acquisition of physiological parameters, and ultimately, data exchange over BLE communication protocol.

The chosen microcontroller is the ultralow-power STM32L432KC (manufactured by STMicroelectronics, Geneva, Switzerland), based on the high-performance Arm Cortex M4 32-bit RISC core operating at frequencies up to 80 MHz, which integrates important features, such as a 12-bit analog-to-digital converter (ADC), USB 2.0 full speed, low-power UART, and two separate I^2^C channels [50]. Its features enable an efficient management of the data reading and exchange processes without latency issues while ensuring ultra-low-power capabilities, essential for a battery-powered device.

The management of peripherals is carried out through three data buses that have been implemented to ensure communication with digital sensors, which occurs through the I^2^C protocol (I^2^C BUS), communication with the BLE module using the UART serial protocol (UART BUS), and, finally, the reading of raw data originating from analog sensors (ADC BUS), respectively. The MAX30102 PPG sensor and the motion detection module MPU6050 together constitute the digital sensor system within the device.

The MAX30102 (manufactured by Analog Devices, Inc., Wilmington, MA, USA) is an integrated sensor designed for acquiring the PPG waveform in reflection mode, which is achieved through a photodetector and two light-emitting diodes operating in the red (660 nm) and in the infrared spectrum (880 nm) [51]. The MAX30102 features a high-resolution (up to 18 bit) low-noise internal ADC, complemented by an ambient light rejection circuit. Both elements contribute to obtain high-quality PPG signals under typical usage conditions, including situations with significant ambient light presence, as illustrated in a previous work [36]. In our application, the MAX30102 has been configured to acquire both wavelengths at a sampling frequency of 1 kHz with a 16-bit ADC resolution. This sampling frequency is adequate for carrying out HRV analyses and PAT computation, since such a high temporal resolution enables the detection of time delays in the order of milliseconds between ECG and PPG waveforms.

The second digital sensor integrated in the system is the MPU6050, which is a complete inertial measurement unit, known for its solid performance in capturing acceleration and angular velocity data [52]. This compact module includes both a three-axis accelerometer and a three-axis gyroscope. In our device, the MPU6050 has been integrated to acquire accurate acceleration signals along the three orthogonal axes. The sampling frequency of this module has also been set to 1 kHz, in order to be the same as that of the PPG sensor and to ensure the highest fidelity in data capture.

The analog sensors block connected to the ADC BUS line consists of ECG and GSR sensors. As shown in the bottom right corner of Figure 1, each sensor can be divided into two main parts: the electrodes, which allow the detection of electrical signals directly on the fingers, and their respective conditioning circuits, which enable the high-quality signal acquisition performed by the MCU’s internal ADC. In detail, the ECG signal is acquired through three electrodes, and unlike all the other recorded signals, it requires the use of fingers from both hands (specifically, the thumb of the left hand and the index and middle fingers of the right hand): the first captures the electrical potential on the phalanx of the left thumb (the same hand in which the device is intended to be worn); the second one serves the same purpose but operates on the right hand index finger; finally, the third electrode is placed in contact with the right hand middle finger and can optionally be used to activate the right leg drive (RLD) circuit, which reduces electrical noise and enhances the quality of the recorded ECG signal. With this spatial arrangement, the electrodes follow the geometry of Einthoven’s Triangle, thus enabling the acquisition of a single-lead ECG (lead I) [53]. The signal detected by the electrodes is then sent as input into the AD8232 chip, an integrated analog front-end designed for the measurement of biopotentials, allowing the extraction, amplification, and filtering of the small biopotentials detected by the electrodes [54]. The AD8232 output is then connected to the ADC BUS line, where the high-quality ECG signals are sampled.

The GSR signal is acquired through two electrodes placed in contact with two diametrically opposite areas of the index finger of the left hand. From a methodological perspective, the measurement of this signal relies on the volt–amperometric method for acquiring skin resistance measurements [38]. By applying an electrical potential to one electrode and measuring the remaining potential in the other one (i.e., the sensing electrode) while also monitoring the current flowing through the electrodes, it becomes possible to compute the resistance value provided by the epidermal tissue. The size of the electrodes and the applied voltage on the finger limit the current density below 10 µA/cm^2^, the threshold indicated by publication recommendations for electrodermal measurements to avoid the possible sweat gland damage [38]. The signal detected by the sensing electrode is then routed to a dedicated conditioning circuit that, through amplification and filtering operations, improves the signal quality. Further details on the analog front-end circuit for GSR acquisition can be found in our previous work [36]. Finally, the output of the conditioning circuit is connected to the ADC BUS line. The acquisition of both analog signal outputs from their respective conditioning circuits is carried out by the internal ADC of the MCU, which has been configured to capture the signals at a resolution of 12 bits and at a sampling frequency of 1 kHz. The synchronization of the acquired data is ensured by the data reading routine, executed by the MCU cyclically every millisecond throughout the duration of the measurements. This routine is executed whenever the MAX30102 completes an acquisition of both PPG signals, as communicated by the latter through an interrupt signal generated by interrupt (INT) pin. Upon the arrival of this signal, the MCU enters the measurement reading routine, reading and storing in a buffer the ECG, GSR, and motion data acquired in the same cycle, respectively, from the MCU internal converter and the MPU6050 module.

The BLE communication protocol has been chosen to remotely transmit the recorded biosignals, since it exhibits an excellent trade-off between transmission speed and energy consumption and is among the most commonly used wireless communication protocols within wearable technologies. For the implementation of BLE communication, the HM-18 module based on Texas Instruments (Dallas, TX, USA) CC2640R2F IC has been utilized, which is controlled by the MCU through the UART BUS line and, once paired, enables data exchange with other BLE devices in proximity.

Finally, as depicted in the top right corner of Figure 1, the entire system is powered by the battery management system (BMS), which primarily comprises a lithium battery managed by a charge controller circuit, and together with LDO regulators provides the voltage levels required by the rest of the components (i.e., MCU and sensors).

Beyond the architecture of the system, relevant considerations about the shape of the device have been taken into account in order to allow the acquisition of high-resolution signals without compromising the compactness and adaptability of the product. A ring-shaped device can satisfy both needs with the drawback of a slightly more complex assembly procedure, due to the use of flexible printed circuits.

**Figure 2:**
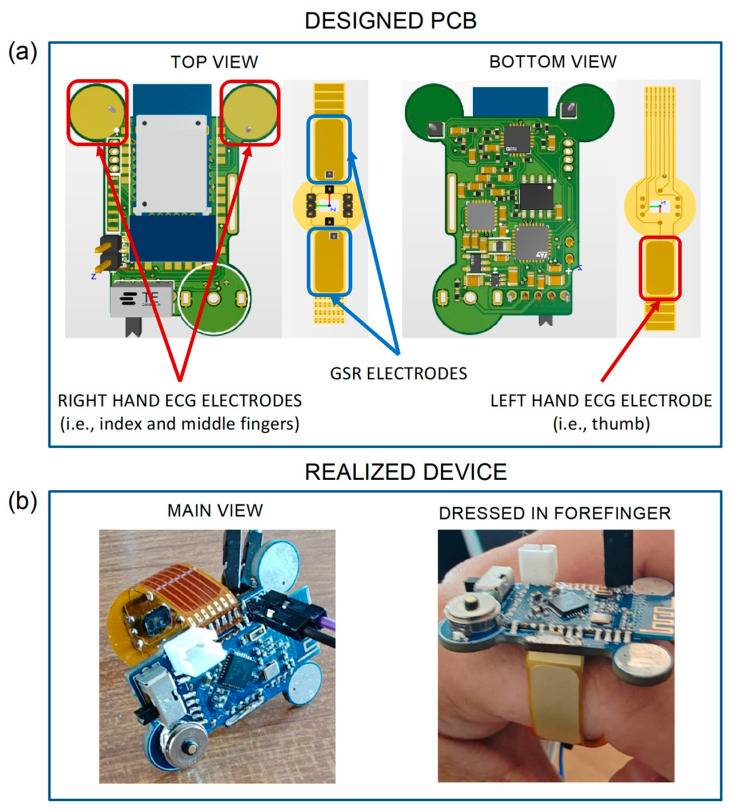
(**a**) Top and bottom views of the rigid PCB (in green) and flexible PCB (in yellow), with the latter hosting the PPG MAX30102 sensor for acquiring the PPG waveform on the underside of the finger. The ECG and GSR electrodes are highlighted with red and blue rectangles, respectively; the fingers used for the acquisition of the ECG signals are indicated in parentheses. (**b**) Photographs of the developed ring-shaped device, in its main view (left) and while being worn on the forefinger.

Figure 2a shows both the top and bottom views of the PCB designed for the development of our device. In detail, the rigid PCB (in green) houses all the above-described components except for the MAX30102 sensor, which is instead integrated into the flexible PCB (in yellow) to acquire the PPG signal on the underside of the finger.

The yellow areas in the top view of the rigid PCB (highlighted by the red squares) represent the first two ECG electrodes, while the third electrode is indicated by the red rectangle in the bottom view of the flexible PCB. The PCB also integrates two GSR electrodes, denoted by the two blue rectangles in the top view. For carrying out the measurements, the proper and good contact between skin and electrodes is an essential requirement. Therefore, all the electrodes on the flexible PCB were fabricated using the electroless nickel immersion gold (ENIG) surface finish process, which is a metal suitable for creating flexible electrodes. Thanks to the gold surface, they behave very similarly to Ag/AgCl electrodes used as a standard for GSR [38,55]. Furthermore, thanks to the ergonomic ring shape of the device, these electrodes can well adhere to the finger, allowing a constant and continuous contact over time. Moreover, in order to improve the ECG electrode usability, two hot air solder leveling (HASL) finished pads are positioned in the forward part of the PCB, which also avoid undesirable contacts between the fingers and other parts of the PCB. The potential problem with the reliability of ECG measures is solved with the proper choice of electrode positions. Similarly, from preliminary tests, it was observed that an 8 mm diameter allows for an adequate contact between the skin and electrodes and reduces inter-subject variability. Particular care was taken to avoid placing components on the top side of the PCB, in order to leave the whole space for the Bluetooth module, and to position BLE antenna outside the PCB in order to ensure the best data transmission performances. The whole circuit has been carefully designed to reduce mutual interferences between analogue’s highly sensitive traces and digital paths; for this reason, a multi-layer routing technique has been employed. The PCB is thought to be produced in a factory with pick and place machines and reflow soldering techniques only. In this way, it has been possible to shrink components with a tenth of a millimeter tolerance. The design has been developed using the CAE software Altium Designer 22 (San Diego, CA, USA).

In Figure 2b, the developed ring-shaped wearable device is shown, in its main view (left panel) and while being worn on the forefinger (right panel). As shown, the main rigid PCB encompasses all the electronic components except for the ones used for PPG acquisition. In the figure, it is possible to note the switch to turn on the device, the Bluetooth module used for wireless communications, the battery connector, the two circle-shaped ECG electrodes on the upper part, and a magnetic two pole connector used for battery charging. Compared to standard USB-C charging solutions, a magnetic connector significantly helps ensuring the compactness on the device; however, a dedicated charger for the device is needed. The black connectors were used for programming purposes only during testing phases; hence, they are not an integral part of the circuit and can be easily removed after the upload of the final version of the firmware. The yellow part is the flexible PCB, which is realized with a 0.2 mm polyimide film, and performs three functions, i.e., guaranteeing a proper wearing of the device on the finger (see Figure 2b), hosting the integrated circuit for PPG acquisition in reflection mode, and providing conductive surfaces usable as electrodes for ECG and GSR acquisitions.

### 2.2. Experimental Protocol

Six healthy subjects (three females, age 27.3 ± 2.9 years) were recruited and subjected to two different measurement protocols designed to simulate various activities typically performed in daily-life scenarios. Each protocol lasted for 12 min and consisted of two phases differentiating between resting and stress conditions.

The first measurement protocol comprised a resting phase, during which the subject was lying on a bed in a supine position (SUPINE) for 6 min, followed by a standing phase (STAND) in which the subject stood upright for another 6 min.

The second measurement protocol required the subject to remain first seated on a chair for 6 min, followed by a walking phase (WALK) lasting 6 min with the subject instead moving at a normal walking speed. In order to standardize the walking speed, a sports treadmill set with a speed of 4 km/h was utilized. This speed was also chosen to minimize as much as possible any small movements of the wearable device, given by the fact that it might tend to slip during walking in case of imperfect adherence to the subject’s finger.

The device was worn by all subjects on the index finger of the left hand, where the recordings of PPG (red and infrared), GSR, and movement signals were acquired. During SUPINE, STAND, and SIT phases, the ECG signal was also acquired by requiring the subjects to put their left-hand thumb and their index and middle fingers of the right hand on the corresponding ECG electrodes, as previously described (see Figure 2a).

### 2.3. Data Processing and Time Series Extraction

An appositely developed MATLAB-based graphical user interface was designed for managing the system and transferring the acquired data to a computer, also enabling real-time visualization and preprocessing of the signals. All the operations described below, including filtering, processing, and signal analysis, were carried out within the MATLAB environment (MATLAB R2022b©, The MathWorks, Inc., Natick, MA, USA).

The raw ECG signal was processed using a zero-phase fourth-order bandpass Butterworth digital filter, with lower and upper cutoff frequencies set at 0.1 Hz and 20 Hz, respectively. Subsequently, a modified version of the Pan–Tompkins algorithm [56] was employed to detect the R peaks in the ECG trace. The PPG signals (both red and infrared) were processed using a zero-phase fourth-order lowpass Butterworth digital filter, with a lower cutoff frequency of 8 Hz. A peak detection algorithm was implemented to identify the maxima and minima of the two signals. Lastly, a zero-phase fourth-order lowpass Butterworth digital filter with a cutoff frequency of 4 Hz was utilized to filter the GSR signal. The acquired data underwent a visual inspection to ensure suitability for subsequent processing and analysis. Accelerometer data were also visually analyzed to proactively identify any motion artifacts that could potentially corrupt the relevant signals.

Afterwards, for each phase of the measurement protocol, according to the standard short-term HRV analysis approach, a temporal sub-window was selected to obtain 300-beat-long time series [29]. From the ECG signal, RR time series were extracted by considering the temporal distance between two consecutive R peaks. Similarly, PP time series were extracted by considering the temporal distance between two consecutive minima of the PPG waveform, for both the red (PP_red_) and infrared (PP_ir_) wavelengths. Starting from the minimum and maximum values extracted from the red and infrared PPG signals, it was possible to calculate the SpO_2_ values using the same methodology (i.e., empirical model and calibration process) implemented in our previous work [36] to which the readers can refer to for further details.

From the literature, it is well known that the infrared PPG signal exhibits better resolution compared to the red PPG one due to the ability of the infrared wavelengths to penetrate deeper into human tissues [57,58]. This is reflected in a more reliable detection of the minima of the PPG waveform, resulting in the extraction of PP time series that are more similar to the RR time series. In this work, this aspect was investigated using the Bland–Altman analysis [59], a useful tool for evaluating the agreement between two measurements. Specifically, the agreement coefficient between the PP_red_ and PP_ir_ time series, as well as between each of them and the RR time series, was obtained for each subject and acquisition phases by using the following formula:

(1) $$Agreement=\frac{1.96\cdot std(x_{1}-x_{2})}{mean((x_{1}+x_{2})/2)}$$

where $x_{1}$ and $x_{2}$ represent the two compared time series. Low and closer to zero values for this index are indicative of a good agreement between a measurement under investigation and a well-established reference gold standard. The agreement measures between PP_ir_ and PP_red_ time series were computed for all the four phases (i.e., SUPINE, STAND, SIT, and WALK phases). On the other hand, the agreement between RR and PP time series was computed only for SUPINE, STAND, and SIT phases and not for the WALK phase, since the ECG signal was not acquired in this last part of the experimental protocol.

In light of the worse quality of red PPG signals, PP_red_ time series have been discarded for further cardiovascular dynamic analysis. Similarly, the PAT time series, calculated by considering the time interval between the R-peak of the ECG and the peak of the PPG waveform within the same cardiac cycle [33], were obtained by using the infrared PPG signal.

The GSR time series were obtained by sampling the GSR signal at the peaks of the infrared PPG waveforms, and the analyses were focused on calculating the mean and standard deviation of the obtained GSR time series. Finally, an estimation of respiratory rate was carried out from the PPG signals, by applying a bandpass filter to the infrared PPG waveforms, based on the knowledge that respiratory variability is typically contained in the high-frequency (HF) band (0.15–0.4 Hz) [37,60]. The respiratory time series were extracted by sampling the reconstructed breathing signal at the peaks of the infrared PPG signal, and then estimating the power spectral density through the weighted covariance method, thereby identifying the respiratory rate. Further details on the used algorithms are reported in a previous work [37], in which the respiratory parameters computed from PPG-reconstructed waveforms were tested against reference breathing signals to analyze cardiorespiratory interactions.

### 2.4. HRV and PAT Analyses

The RR, PP_ir_, and PAT time series were analyzed to extract indices reflecting physiological changes in response to the different phases of the acquisition protocol.

The classical HRV time-domain indices were calculated, i.e., the average (MEAN), standard deviation of the normal-to-normal intervals (SDNN), and root mean square of successive differences between normal heartbeats (RMSSD) on RR and PP_ir_ time series (in the latter case, we refer to pulse rate variability, PRV) [27]. Analogously, the time-domain indices of MEAN and standard deviation (STD) were computed on the PAT time series.

Before performing frequency-domain and information-theoretic analysis, physiological time series were preprocessed applying a high-pass autoregressive filter (with a cutoff frequency of 0.0156 Hz) and normalizing the filtered series to zero mean and unit variance. The non-parametric Blackman–Tukey method (Hamming window, bandwidth of 0.04 Hz) was applied to obtain the power spectrum for each subject and condition of RR, PP_ir_, and PAT time series [61]. The low-frequency (LF) and HF power contents were evaluated by integrating the distribution in the ranges of 0.04–0.15 Hz and 0.15–0.4 Hz, respectively [27]. These power values are generally used to obtain the ratio between the LF and HF contents. Although still debated, this index gives insight into the sympathovagal balance in HRV analysis [27]. Conversely, its role in the analysis of PAT variability is not clear and seems to be unrelated to the ANS regulatory activity [62].

Finally, the conditional entropy (CE) measure was estimated to characterize the time series complexity in terms of its irregularity and unpredictability, quantifying the residual uncertainty about the current state of the process remaining when past dynamics are known [63]. Specifically, this index decreases as the predictability of the series increases and reaches zero for fully predictable dynamics. Under the hypothesis of Gaussianity and stationarity for the process X, this index was computed as follows:

(2) $$CE=\frac{1}{2}\ln\left(2\pi e{\hat{\sigma }}_{U}^{2}\right)$$

where ${\hat{\sigma }}_{U}^{2}$ is the estimated variance of the prediction error U of the linear regression describing the evolution of the process over time, i.e., $X_{n}=\sum_{k=1}^{m}a_{k}X_{n-k}+U_{n}$ with $a_{k}$ the regression coefficient related to lag k. In this work, the order of the AR model used to the describe the physiological processes was set to *m* = 2, and the ordinary least squares method [64] was used to identify the variance ${\hat{\sigma }}_{U}^{2}$ starting from the regression coefficients.

### 2.5. Statistical Analysis

In this work, a statistical analysis was performed to assess the feasibility of using the above-described indices to discriminate changes between the different physiological conditions. Bootstrap data analysis was employed to assess the statistical significance of the results, generating a distribution of values for each subject, phase acquisition protocol, and feature computed on RR, PP_ir_, and PAT time series. Specifically, considering what is reported in the literature about the ultra-short-term analysis [65,66,67], 100 data windows of 120 samples each were randomly extracted from the original 300-point time series, and then time, frequency, and information domain indices were computed for each of the surrogate data. This approach allows to obtain for each subject a distribution of the evaluated index in the different acquisition phases, thus becoming statistically comparable. Specifically, a parametric Student *t*-test for unpaired data was applied to compare the distributions of the indices evaluated on surrogates obtained in the SUPINE and STAND positions, as well as in the SIT and WALK conditions (in this case, taking into account only the PP_ir_ time series indices). For all the comparisons, the significance level was set at *p* < 0.05.

## 3. Results

**Figure 3:**
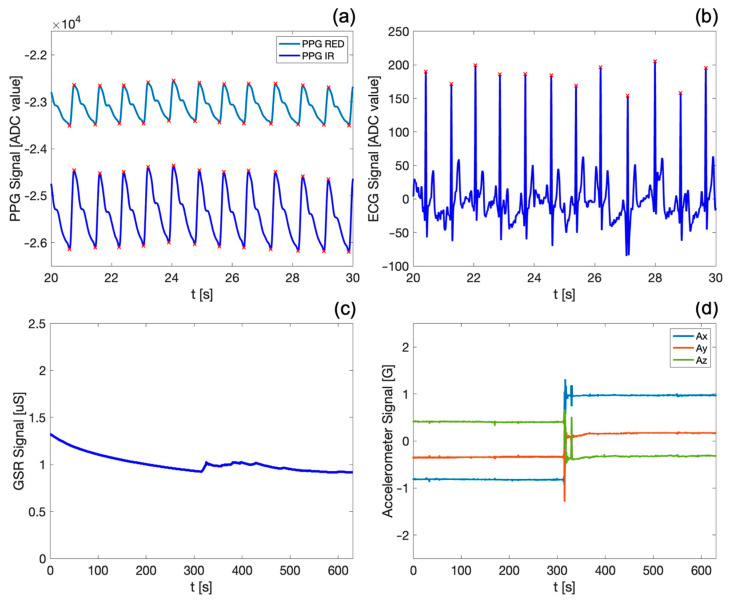
Exemplary signals acquired on a subject during the first measurement protocol: (**a**) PPG (both red and infrared) and (**b**) ECG signals in a 10 s time window and (**c**) GSR and (**d**) motion signals over the entire duration of the protocol. In (**a**,**b**), red crosses indicate PPG maxima/minima and ECG R peaks, respectively.

In this section, the results of the acquisitions carried out using the ring-shaped device during the measurement protocols are reported. Figure 3 shows the PPG waveforms (both red and infrared) and the ECG, GSR, and motion (i.e., spatial acceleration along) signals acquired by the device during the SUPINE–STAND measurement protocol.

In particular, Figure 3a,b show the detail of a 10 s acquisition window, allowing the visualization and qualitative appreciation of the high fidelity of the acquired biosignals. Both PPG waveforms exhibit the typical morphology reported in the literature [17,20], with the dicrotic notch clearly visible; the end of the systolic phase and of the diastolic phase (indicated by the maximum and the minimum of the PPG signal, respectively) are marked by red crosses. It is noteworthy that the PPG infrared signal exhibits a better quality, since the infrared wavelengths penetrate more deeply into the body skin and tissues [57,58], as previously discussed. Similarly, the ECG signal appears stable and well defined, with the R peaks (also marked by red crosses in Figure 3b) easily distinct from other points in the QRS complex.

Figure 3c,d depict exemplary waveforms of the skin conductance (i.e., GSR) and motion signals over the entire duration of the first measurement protocol, which lasts approximately 12 min. The GSR signal shows a slight decrease over time, confirming that the subject was in a resting state during the first phase of the measurement protocol. Furthermore, the trend and values of the GSR signal on the fingers align with those found in the reference literature [38,68] for similar physiological conditions. The variations in accelerometric signals in the x, y, and z axes (Figure 3d) allow the identification of the subject’s position and of sudden voluntary or involuntary movements, substantially contributing to the obtainment of further information about the subject’s motor activity during the measurement protocol. Indeed, an abrupt variation in the accelerometric data can be observed, indicating the actual change in the subject’s position from SUPINE to STAND, which occurs precisely at the halfway point of the first protocol. The GSR signal (Figure 3c) also identifies this transition, as evidenced by a sudden increase in sweating corresponding to the phase change. It is worth underlining that the visualization of all the biosignals acquired by the system is of great assistance in highlighting capabilities and functionalities of our device, especially considering that all these traces are acquired synchronously on the same body district.

**Figure 4:**
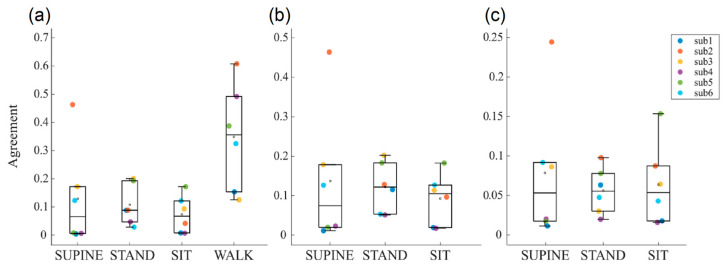
Boxplot distributions of agreement index of the Bland–Altman method computed between (**a**) PP_ir_ and PP_red_, (**b**) PP_red_ and RR, and (**c**) PP_ir_ and RR, evaluated across the six subjects during the four phases (i.e., SUPINE, STAND, SIT, and WALK) of the two measurement protocols.

Figure 4 shows the boxplot distributions of the agreement index of the Bland–Altman method computed between RR and PP time series, respectively, extracted from the ECG trace and the two PPG signals. These analyses were conducted to assess the reliability of the interbeat interval time series extracted from the two PPG traces, compared to ECG reference. Furthermore, this comparison is also crucial to assess the reliability of the time series during the different phases of the protocol, in order to understand whether motor activity might lead to the corruption of the computed time series values.

The agreement between PP_ir_ and PP_red_ extracted during the two measurement protocols is shown in Figure 4a. A very good agreement is observed for almost all subjects in phases without motor activity (i.e., SUPINE, STAND, and SIT phases), with values ranging between 0 and 0.2, except for the subject 2 during the SUPINE phase. Conversely, in the presence of motor activity, there is a noticeable deterioration in the agreement, with an average value of 0.35 and an increased dispersion of values among subjects. This finding is expected, as physical activity leads to worsened measurement conditions, primarily due to the presence of motion artifacts resulting in a reduced quality of the acquired physiological signals, with the PPG signal being particularly affected. This leads us to consider the physiological indices extracted from PPG signals, e.g., blood oxygen saturation and respiration rate, to be less reliable during this phase. Figure 4b,c show the agreements obtained by, respectively, comparing PP_red_ and PP_ir_ with RR taken as the reference. Even if similar distributions can be observed across the acquisition phases, it is interesting to note how agreement values range between 0 and 0.2 for PP_red_ and are halved for PP_ir_, thus confirming once again the overall better quality of the infrared PPG signals.

Starting from the above-described results on the quality of the acquired signals, the subsequent analyses were focused on the extraction of most physiological indices from the PP_ir_ time series only. The evaluation of the blood oxygenation makes an exception, requiring both PPG waveforms.

Table 2 presents the physiological indices (expressed as mean value ± standard deviation) extracted in the time, frequency, and information domains from 300-point PP_ir_, RR, and PAT time series, obtained from the six subjects during the two measurement protocols. The reported values during the SUPINE, STAND, and SIT phases, and for some of the time series even during the WALK phase, are consistently in line with what is typically reported in the literature, both for resting situations that are followed by the transition to more stressful physiological conditions [68,69,70]. In detail, the heart rate is lower in resting phases and increases during the following two physiological conditions. This response reflects the level of activation of the ANS as also indicated by an average decrease in the SDNN and RMSSD indices with STAND, and contrarily an increase during SIT (except for RMSSD_PPir_). Moreover, as expected, the LF/HF ratio increases during the other phases of the protocol if compared to REST. CE values are lower during the STAND phase, indicating higher regularity and predictability of cardiac dynamics.

All these results reflect the well-known prevalence of the parasympathetic branch activity in a resting condition and the sympathetic activation occurring during stress states. In general, different considerations can be made for the measurements obtained during the WALK phase. Indeed, the consistent presence of motion artifacts leads to a degradation of the PPG signal and, consequently, of the PP_ir_ time series. This results in a loss of reliability in the extracted physiological indices, since it is possible to observe an unexpected increase in SDNN, RMSSD, and LF/HF values, accompanied by an increase in CE, indicating an augmentation in the complexity of the PP_ir_ time series. The worsening of the quality of the PPG waveform during WALK is also evidenced by a relevant increase in the variability of SDNN and RMSSD across subjects.

The achieved results on the PAT time series are also supported by previous studies [62] that reported a decrease in the time of arrival of the pressure wave at the body periphery and an increase in its variability during physical stress. Contrarily, no supporting results have been achieved in the literature about the reported findings in the frequency domain, which show an increase in the LF/HF ratio during STAND and SIT.

The modification in the ANS activity is also confirmed by the GSR values computed from the GSR time series, which are lower during resting conditions and higher during stress, especially during the transition from SIT to WALK when the GSR triples its value. Similarly, respiration rate particularly increases during WALK, as expected. The SpO_2_ index is in line with the expected values (97–99%) during resting conditions, while the presence of artifacts degrades the PPG signal up to severely limiting its use for calculating blood oxygenation levels during non-static measurement conditions, given that the agreement between the two PPG signals worsens excessively (cf. Figure 4a).

**Figure 5:**
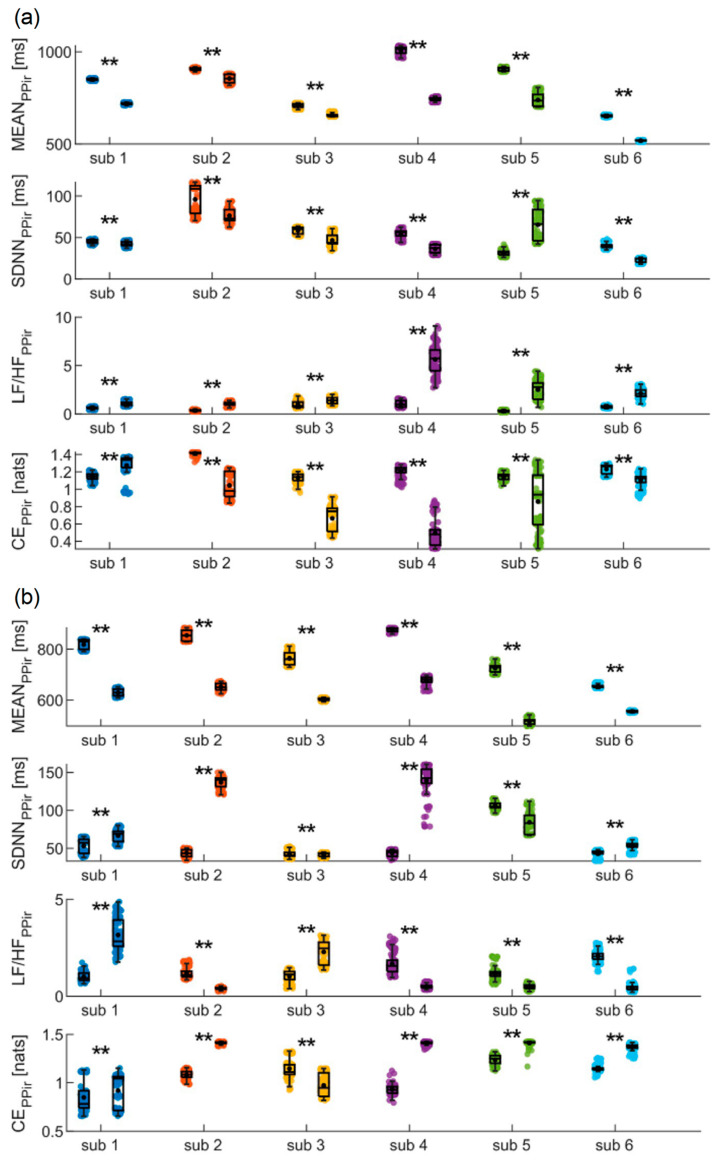
Results of the PP_ir_ time series analysis in time, frequency, and information domains of MEAN, SDNN, LF/HF, and CE evaluated on the six subjects during (**a**) the SUPINE–STAND and (**b**) the SIT–WALK measurement protocols. Statistical analyses were performed between the two different phases considering one hundred sub-windows of 120 points randomly extracted from the PP_ir_ time series. Statistical test: Student’s *t*-test, **, *p* < 0.001, SUPINE vs. STAND.

**Figure 6:**
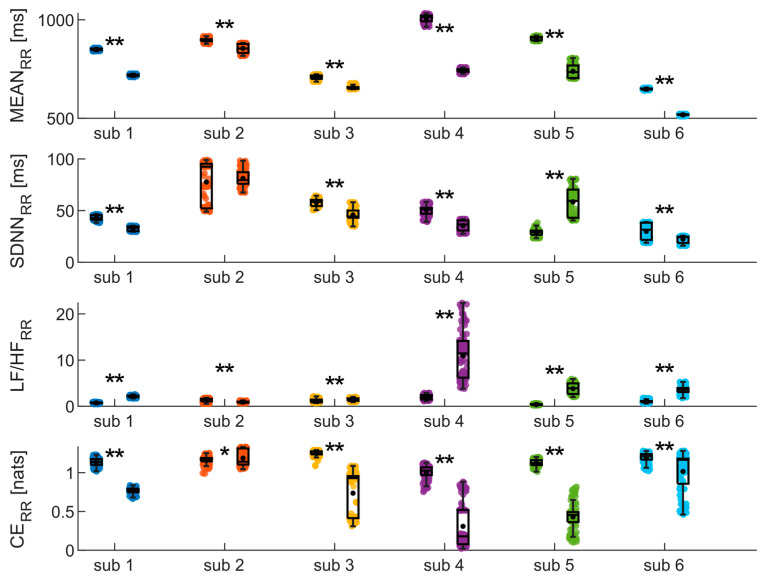
Results of time, frequency, and information domains analysis on the RR time series, evaluated on the six subjects during the SUPINE–STAND measurement protocol. Statistical analyses were performed considering one hundred sub-windows of 120 points randomly extracted from the RR time series. Statistical test: Student’s *t*-test; *, *p* < 0.05, **, *p* < 0.001, SUPINE vs. STAND.

**Figure 7:**
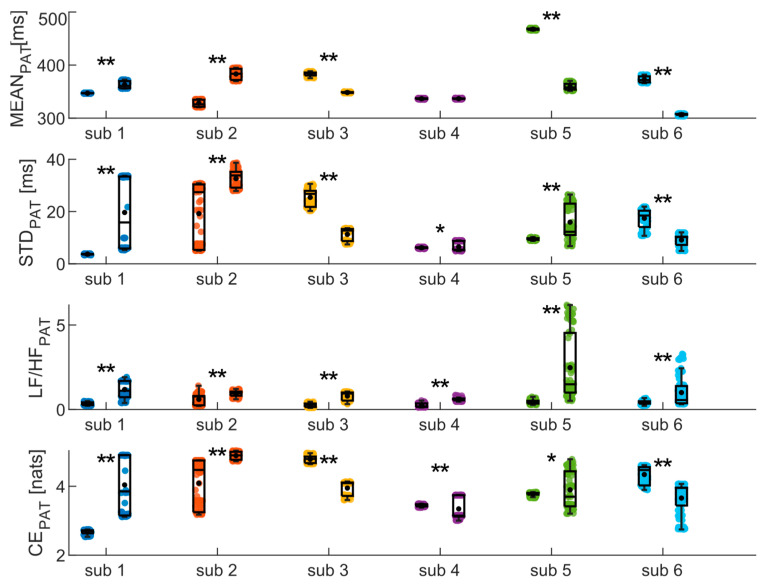
Results of the analysis of PAT time series in time, frequency, and information domains, evaluated on the six subjects during the SUPINE–STAND measurement protocol. Statistical analyses were performed considering one hundred sub-windows of 120 points randomly extracted from the starting PAT time series. Statistical test: Student’s *t*-test; *, *p* < 0.05, **, *p* < 0.001, SUPINE vs. STAND.

Figure 5, Figure 6 and Figure 7 depict the results of the bootstrap analysis performed on the time, frequency, and information domains indices computed for the PP_ir_, RR and PAT time series, evaluated individually for each subject. Statistical analyses were performed on the distributions obtained in the SUPINE and STAND positions, as well as SIT and WALK positions for the PP_ir_ time series, in order to prove on a single-subject basis the capability of using the device for discriminating physiological changes.

The results of the analyses conducted on PP_ir_ during the SUPINE and STAND phases are shown in Figure 5a. Both time-domain indices (i.e., MEAN and SDNN) decrease significantly from the first to the second phase for all the subjects except the fifth, showing instead a significant increase in SDNN. Frequency analysis reveals that in all subjects a statistically significant increase in the LF/HF ratio is detected. Finally, CE measures undergo a significant decrease for all subjects except the first, for whom a significant increase is observed from the SUPINE to the STAND phase. In Figure 5b, the results obtained for the same time series during the SIT and WALK phases are reported. In this case, a significant decrease in MEAN values is observed for all subjects as well, while a statistical increase in SDNN values is observed for all subjects except the fifth, showing instead a significant decrease. The LF/HF ratio significantly decreases for four subjects, while a statistical increase is observed for the other two. Finally, CE significantly increases in all subjects except the first, for whom a statistical decrease is instead reported.

The results of the analyses conducted on the RR time series during SUPINE and STAND are depicted in Figure 6. They confirm most of the findings obtained from the analysis of the PP_ir_ time series, as nearly identical trends in the indices are reported compared to those highlighted with regard to Figure 5a. Indeed, also in this case, both MEAN and SDNN undergo a significant decrease for all subjects, with the only difference being that, in this instance, no statistical difference is detected in the SDNN values for the second subject between the SUPINE and STAND phases. The trend is also confirmed for the LF/HF ratio, where a significant increase is once again observed for all subjects. Finally, CE shows a statistically significant decrease in all subjects.

Finally, the same analyses were conducted on the PAT time series, yielding results highly dependent on the subject (as depicted in Figure 7)). Specifically, significant increase and decrease in MEAN are observed for two and three subjects, respectively, while no difference is detected for the fourth subject. Similarly, CE measures indicate an increase between phases for three subjects and a decrease for the other three. The results of the frequency analysis, on the other hand, show a significant increase in the LF/HF ratio values for all subjects.

## 4. Discussion

The aim of this work is to present a novel multiparametric wearable device that enables multiparametric acquisition of high-quality biosignals. The analysis of multiple physiological indices extracted from the various biosignals allows to increase knowledge about the physiological response of the body during different conditions of physical activity typically experienced in daily routines.

The design of the electronic board was made with a particular focus on signal integrity, by splitting the analog and digital parts into separate sections, since power and two ground planes connected in a star configuration are used. The position of the BLE antenna was also studied for preventing electromagnetic interferences (EMI), which could potentially affect other parts of the circuit. Along with the careful selection of integrated biomedical sensors, this led to the development of a very compact wearable device thanks to the reduced weight and size, without compromising electrical performances [71]. The device allows the acquisition of key physiological parameters directly from the index finger, providing clear advantages in terms of usability and user comfort. In particular, the sensors (i.e., the ECG and GSR electrodes) and the inertial module have been suitably placed in strategic points on the fingers of the hand (Figure 2), enabling the acquisition of the biosignals in a simple and comfortable manner. Furthermore, the system architecture (Figure 1) has been appositely designed to enable the synchronous high-resolution acquisition of the recorded biosignals (Figure 3) at a sampling frequency of 1 kHz. To the best of our knowledge, such a higher sampling rate, together with the synchronous acquisition of GSR signal, constitutes the main advantage of our device compared to the currently available wearable technologies (Table 1).

The results obtained from the agreement measurements between cardiovascular signals (i.e., PPG and ECG ones) have allowed considering the PP time series as surrogates for the RR ones during stationary physiological states (Figure 4b,c). This is a remarkably interesting result, given that it is well known in the literature that the ECG signal is more challenging to acquire due to its measurement methodology which, in the case of wearable devices like ours, requires the use of both hands throughout the entire measurement duration [46,48]. This also paves the way for the deployment of the PPG signal as a surrogate for ECG in situations where it is impractical to require the user to engage both hands during recordings.

The findings of the short-term analyses summarized in Table 2 highlight the feasibility of employing the indices extracted from our device to detect the physiological response of the body following a postural change even using less amounts of data (i.e., considering 120-point time windows). Moreover, the single-subject analysis performed using combined bootstrap method with ultra-short-term HRV indices shows how the results achieved using PP and RR time series are in agreement and almost consistent among subjects. Indeed, the results of PRV (Figure 5a) and HRV (Figure 6) analyses, evaluated during the SUPINE–STAND protocol, agree with the widely documented evidence in the literature regarding the variations in the physiological indices between rest and stress conditions [24,65,72,73,74]. Specifically, a decrease in MEAN and SDNN and an increase in LF/HF and CE can be observed, trends which have been widely put in relation to a shift in the sympathovagal balance in favor of the sympathetic branch, which is typical during transitions from resting to stress physiological conditions [75,76]. The activity of the SNS also causes an augmented activity of sweat glands, resulting in an increase in the epidermal sweat: this physiological mechanism is detected by the GSR signal, whose values are higher during stress conditions (Table 2). Additionally, it is well established that the increase in the sympathetic activity caused by orthostatic stress leads to a reduction in cardiac dynamics, as evidenced by the significant decrease in CE from the rest to stress phase [32,77,78]. Regarding the ultra-short-term PRV analysis conducted during the SIT–WALK protocol (Figure 5b), it is possible to observe the same results highlighted by the indices extracted for the short-term analysis (Table 2). A decrease in the MEAN index is reported in all subjects, in line with typical trends of this parameter during stress situations. On the other hand, there are unexpected variations in SDNN and LF/HF, which show different trends between subjects. Furthermore, there is a significant increase in CE in all subjects, indicating an increase in the complexity of the PP time series during the WALK phase. The analyses conducted on the PAT time series (Figure 7) report the average values of the extracted indices that align with those reported in the literature for healthy subjects in both resting and orthostatic stress conditions [35,62,70]. Lastly, our findings highlight a strong deterioration of the agreement levels between the two PPG signals, especially during the WALK phase (Figure 4a), confirming the well-known fact that the PPG signal is overly sensitive to motion artifacts typically encountered during physical activity.

However, on the one hand, these results underscore the potential of the multiparametric acquisition capabilities of our device, and at the same time, they highlight the well-known limitations of the reliability of PPG signals extracted during non-stationary conditions like walk (Figure 4a) [79,80,81], which entails the deterioration of the signal and consequently of the extracted physiological indices, as previously discussed. These findings strongly encourage the future implementation of motion artifact correction algorithms to allow for a more accurate detection of physiological parameters even during physical activities [40,41]. In this regard, the embedded inertial module, which has been integrated into the device to enable the detection of voluntary and involuntary movements, is fundamental for providing useful data for a future use by artifact correction algorithms. In this way, it will be possible to automatically correct the noisy PPG signal by exploiting the accelerometer data, enhancing the reliability of physiological indices extracted during non-stationary conditions.

## 5. Conclusions

In this work, a novel wearable biomedical device has been presented and discussed, enabling the synchronous acquisition of PPG, ECG, GSR, and motion signals directly on the fingers. The features of the developed device, including its compact size, user-friendly simplicity, and the capability to continuously and non-invasively acquire key physiological parameters, along with the promising results from the first measurement campaign, position it as a potential tool for monitoring individuals during their daily routines. Indeed, preliminary measurement results obtained during various physical activity conditions demonstrated that our wearable device can be successfully employed to monitor and discriminate among different physiological states. The combined analysis of cardiovascular variability indices in the time, frequency, and information domains and of the acquired GSR signals allowed the assessment of the autonomic tone and especially the activation of the sympathetic branch of ANS, thereby providing insights into individual emotional arousal.

Furthermore, the comparison of PRV indices with HRV indices, both extracted from the wearable device, yielded promising results that suggest the validation of PRV indices under stationary physical conditions (i.e., in the absence of motor activity). Our results also confirmed the limitations, widely acknowledged in the literature, regarding the degradation of the PPG signal in the presence of motion artifacts. This finding encourages the implementation of algorithms for the removal and correction of motion artifacts in situations involving continuous physical activity.

Future developments will have to primarily focus on a complete validation of the system using reference patient simulator devices. Concurrently, a future step will be the optimization of the device in terms of weight, size, and shape (e.g., using a microcontroller with embedded Bluetooth module). Additionally, the use of different integrated sensor and power supply technologies will be explored, to further enhance the device performance in terms of signal resolution and energy efficiency (e.g., implementing inductive battery charging) [82,83,84]. Additional measurement campaigns should also be performed, primarily aimed at detecting the physiological states in a larger number of subjects under different physical activity conditions. Another important future development should involve the implementation of the signal processing algorithms directly within the firmware of the device, enabling the real-time computation and extraction of the physiological indices. Finally, the implementation of motion artifact correction algorithms would allow the use of the device in situations involving motor activities, i.e., for fitness applications.

## Figures and Tables

**Table 1:** Comparison of the main wearable devices available on the market in terms of design, recorded biosignals, features and computed physiological indices, and sampling rate [44,45,46,49].

Name	Type of Device	Biosignals	Features and Physiological Indices	Sampling Rate
Apple Watch	Wristband/smartwatch	PPG, ECG, wrist temperature and motion	SpO_2_ levels, HRV, sleep and physical activity tracking, fall recognition and atrial fibrillation detection	500 Hz
Galaxy Watch	Wristband/smartwatch	PPG, ECG, wrist temperature and motion	SpO_2_ levels, HR, sleep and physical activity tracking	25 Hz
Oura Ring	Ring	PPG, finger temperature and motion	SpO_2_ levels, HRV, sleep and physical activity tracking, respiratory rate	250 Hz
Circul+	Ring	PPG, ECG, finger temperature and motion	SpO_2_ levels, HRV, sleep and physical activity tracking, blood pressure	100 Hz

**Table 2:** Results of short-term analysis of PRV, HRV, PAT, GSR, blood oxygen saturation level, and respiration rate (RESP), during the different phases of the measurement protocol and averaged across the six subjects. The values are expressed as mean ± standard deviation.

Measure/Phase	SUPINE	STAND	SIT	WALK
HR_Ppir_ [bpm]	73 ± 12	87 ± 16	77 ± 8	100 ± 10
SDNN_Ppir_ [ms]	55.55 ± 20.94	51.10 ± 21.26	58.65 ± 25.05	85.22 ± 42.08
RMSSD_Ppir_ [ms]	57.47 ± 37.57	38.46 ± 17.95	47.16 ± 25.19	109.94 ± 72.57
LF/HF_Ppir_	0.68 ± 0.30	2.12 ± 1.94	1.30 ± 0.44	1.17 ± 1.10
CE_Ppir_ [nats]	1.20 ± 0.12	0.94 ± 0.31	1.07 ± 0.10	1.27 ± 0.23
HR_RR_ [bpm]	74 ± 12	87 ± 16	77 ± 8	-
SDNN_RR_ [ms]	51.53 ± 20.14	48.68 ± 21.91	59.48 ± 24.96	-
RMSSD_RR_ [ms]	44.55 ± 19.79	31.31 ± 21.63	50.14 ± 26.06	-
LF/HF_RR_	1.05 ± 0.54	3.41 ± 3.55	1.46 ± 0.93	-
CE_RR_	1.13 ± 0.12	0.76 ± 0.34	1.08 ± 0.21	-
MEAN_PAT_ [ms]	372.60 ± 51.01	349.92 ± 26.47	396.31 ± 60.71	-
STD_PAT_ [ms]	14.49 ± 9.26	17.25 ± 9.57	19.66 ± 12.38	-
LF/HF_PAT_	0.39 ± 0.14	1.03 ± 0.44	1.08 ± 0.24	-
CE_PAT_ [nats]	3.92 ± 0.80	4.15 ± 0.51	4.07 ± 0.59	-
GSR [μS]	0.80 ± 0.21	0.86 ± 0.14	1.11 ± 0.39	3.17 ± 1.04
SpO_2_ [%]	97.8 ± 1.6	98.7 ± 1.7	99.9 ± 0.19	88.05 ± 21.11
RESP [Hz]	0.27 ± 0.04	0.23 ± 0.06	0.25 ± 0.07	0.32 ± 0.03

## Data Availability

The data that support the findings of this study are available upon request from the authors.
